# A Descriptive Chemical Composition of Concentrated Bud Macerates through an Optimized SPE-HPLC-UV-MS^2^ Method—Application to *Alnus glutinosa*, *Ribes nigrum*, *Rosa canina*, *Rosmarinus officinalis* and *Tilia tomentosa*

**DOI:** 10.3390/plants11020144

**Published:** 2022-01-06

**Authors:** Thomas Charpentier, Séverine Boisard, Anne-Marie Le Ray, Dimitri Bréard, Amélie Chabrier, Hélène Esselin, David Guilet, Christophe Ripoll, Pascal Richomme

**Affiliations:** 1 EA921 SONAS, SFR4207 QUASAV, Campus du Vegetal, University of Angers, 49070 Beaucouzé, France; thomas.charpentier@univ-angers.fr (T.C.); severine.boisard@univ-angers.fr (S.B.); dimitri.breard@univ-angers.fr (D.B.); david.guilet@univ-angers.fr (D.G.); pascal.richomme@univ-angers.fr (P.R.); 2 Natinov, ZA Montendre, St Lézin, 49120 Chemillé en Anjou, France; amelie.chabrier@natinov.com (A.C.); christophe.ripoll@natinov.com (C.R.)

**Keywords:** concentrated bud macerates, HPLC-UV-MS, flavonoid content, antioxidant activity

## Abstract

Concentrated bud macerates (CBMs) are obtained from meristematic tissues such as buds and young shoots by maceration in a solvent composed of glycerin, water and ethanol (1/1/1/, *v*/*v*). Their traditional utilization in gemmotherapy has gained interest in the past years, and the knowledge of their chemical characterization can provide commercial arguments, particularly to secure their quality control. Therefore, an optimized method for phytochemical analysis including glycerol removal by a preliminary solid phase extraction (SPE) followed by compound identification using high performance liquid chromatography coupled with ultra-violet and tandem mass detectors (HPLC-UV-MS^2^) was developed. This method was applied on 5 CBMs obtained from *Alnus glutinosa*, *Ribes* *nigrum*, *Rosmarinus officinalis*, *Rosa canina* and *Tilia tomentosa* in order to determinate their chemical composition. Their antioxidant effects were also investigated by radical scavenging activity assays (DPPH and ORAC). Glycerol removal improved the resolution of HPLC chemical profiles and allowed us to perform TLC antioxidant screening. Our approach permitted the identification of 57 compounds distributed in eight major classes, three of them being common to all macerates including nucleosides, phenolic acids and glycosylated flavonoids. Quantification of the later class as a rutin equivalent (RE) showed a great disparity between *Rosa canina* macerate (809 mg RE/L), and the other ones (from 175 to 470 mg RE/L). DPPH and ORAC assays confirmed the great activity of *Rosa canina* (4857 and 6479 μmol TE/g of dry matter, respectively). Finally, phytochemical and antioxidant analysis of CBMs strengthened their phytomedicinal interest in the gemmotherapy field.

## 1. Introduction

Bud macerates are obtained from meristematic tissues, such as buds and young shoots, as a solution of glycerin, water and alcohol. These macerates are used in gemmotherapy, which is a popular phytomedicine in European countries [1]. Their utilization in gemmotherapy is supported by a specific and diversified chemical composition, mainly due to the unusual part of plant and extraction solvent. Bud macerates are also described to possess a wide range of biological activities [2,3,4]. However, only a few analytical studies describe their composition, and they were associated with a limited number of different raw materials (Table 1) including *Alnus glutinosa* (L.) Gaertn, *Carpinus betulus* L., *Castanea* spp.: *C. sativa* Mill., *C. crenata* Siebold and Zucc. and *C. sativa* x *crenata*, *Cornus mas* L., *Corylus avellana* L., *Ficus carica* L. and *F. carica* SSP *Dottato*, *Fraxinus excelsior* L., *Juglans regia* L., *Larix decidua* Mill., *Pinus montana* Mill., *Quercus petraea* (Matt.) Liebl., *Ribes nigrum* L., *Rubus* spp.: *R. idaeus* L., *R. ulmifolius* L. or Schoot, *Rosa canina* L., *Salix caprea* L., *Tilia* spp.: *T. tomentosa* Moench., *T. vulgaris* Hayne and *Vitis vinifera* [5,6,7,8,9,10,11,12,13,14,15,16,17,18,19].

Different extraction protocols for bud macerates are described in the literature. Generally, the fresh raw material is submitted to a cold maceration for a long extraction time (minimum 21 days and up to 3 months) with a dry weight/solvent ratio of 5%. The solvent is composed of water, ethanol and glycerol with proportion varying from 0/1/1 to 10/10/1 [5,6,7,8,9,10,11,12,13,14,15,16,17,18,19]. In the case of the French Pharmacopeia protocol, the macerate is further diluted to obtain a 1DH (“Décimale Hahnemannienne”) extract. Recently, accelerated techniques such as pulsed-assisted ultrasound-extraction were proposed as quick, green and alternative processes [6,10,18].

Turrini et al. [6] recently concluded that the polyphenol content of bud derivatives is strongly influenced by manufacturing processes whose parameters are often not strictly defined (for example, extraction solvent ratios, raw material/solvent mixture percentage and extraction time) and thus affect the final composition of bud macerates.

In addition, different classes of metabolites could be identified in meristematic tissues bud macerates: sugars, vitamins, vegetal hormones, amino and nucleic acids, mineral salts as well as glycosylated flavonoids or aglycones, phenolic acids derivatives [16], anthocyanidols [20], mono and sesquiterpenes, saponins and hydrosoluble tannins [13,16,19,21]. This diversity of chemical classes is due to many factors such as cultivar [21], crop itinerary, chemotypes, harvesting time [22], environment [16,23], year of collection [24] and technical conditions. Therefore, the accurate analysis of phytochemicals in bud macerates is of crucial importance.

The present study focused on the phytochemical analysis of five concentrated bud macerates (CBMs) obtained by a cold maceration of fresh plant (1/20 plant, dry weight/solvent) in ethanol/glycerol/water (1/1/1) for 21 days. They were obtained from *Alnus glutinosa* (L.) Gaertn (*Ag*), *Ribes nigrum* L. (*Rn*), *Rosa canina* L. (*Rc*), *Rosmarinus officinalis* L. (*Ro*) and *Tilia tomentosa* M. (*Tt*) and are sold in Europe as food supplements. Bud macerates of *Ag*, *Rc*, *Rn* and *Tt* were already studied for their chemical composition. The presence of flavonols, hydroxycinnamic acids, benzoic acids, catechins, tannins and ellagic acid derivatives was highlighted [5,12,16,19]. Bud macerate obtained from *Ro* is here studied for the first time.

As usually performed, phytochemical profiling was achieved using HPLC-DAD-ESI-MS in comparison with standards and literature data. Moreover, a quick and preliminary solid phase extraction (SPE) was developed and applied to samples to eliminate a great part of glycerol, which is responsible of low-resolution chemical profiles. Flavonoids were part of the main compounds identified in all extracts; therefore, they were quantified as rutin equivalents by HPLC-DAD. As flavonoids are generally associated with strong antioxidant activities, an HPTLC analysis using DPPH as a revelation reagent was performed to confirm the presence of antioxidant compounds. Finally, the antioxidant capacity of the five CBMs was evaluated using DPPH and ORAC assays.

## 2. Results and Discussion

### 2.1. Application of a Solid Phase Extraction Procedure

**Figure 1:**
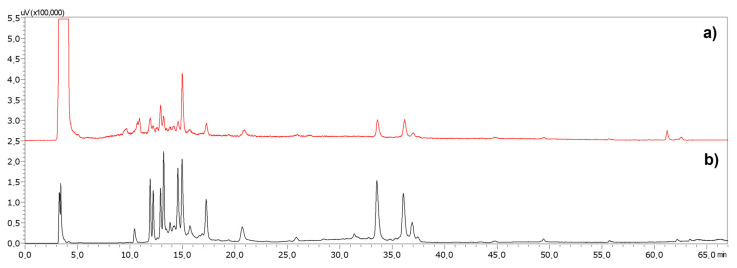
HPLC-ELSD profile of *Rosa canina* concentrated bud macerate before (**a**) and after (**b**) solid phase extraction (SPE).

The presence of glycerin in CBMs affects the resolution of the chemical profiles and avoid the calculation of extraction yields. Therefore, a procedure was developed and applied to all CBMs and the effective elimination of glycerol by SPE was then monitored by HPLC-ELSD. Figure 1 shows, as an example, the *Rc* extract profile before and after glycerol elimination. Based on AUC values, a 9-fold decrease was observed. In the TR 2.5–5.0 min range and when compared with total peak area values, signals associated with glycerol (as well as sugars, organic acids and vitamins) are respectively reduced to 7.8% (*Rc*), 15.5% (*Ag*), 30.4% (*Rn*), 33.8% (*Ro*) and 58.2% (*Tt*), leading to high-quality chromatograms. The use of this method could be particularly useful for the quality control of bud macerates by HPLC fingerprinting.

Extraction yields (m/v %) were then calculated after glycerol elimination. They appeared to range between 0.3 and 0.8% (0.26 ± 0.02% for *Tt*, 0.48 ± 0.02% for *Ro*, 0.49 ± 0.02% for *Rn*, 0.66 ± 0.12% for *Ag* and 0.76 ± 0.07% for *Rc*).

### 2.2. Phytochemical Analysis of Different Concentrated Bud Macerates (CBMs)

**Figure 2:**
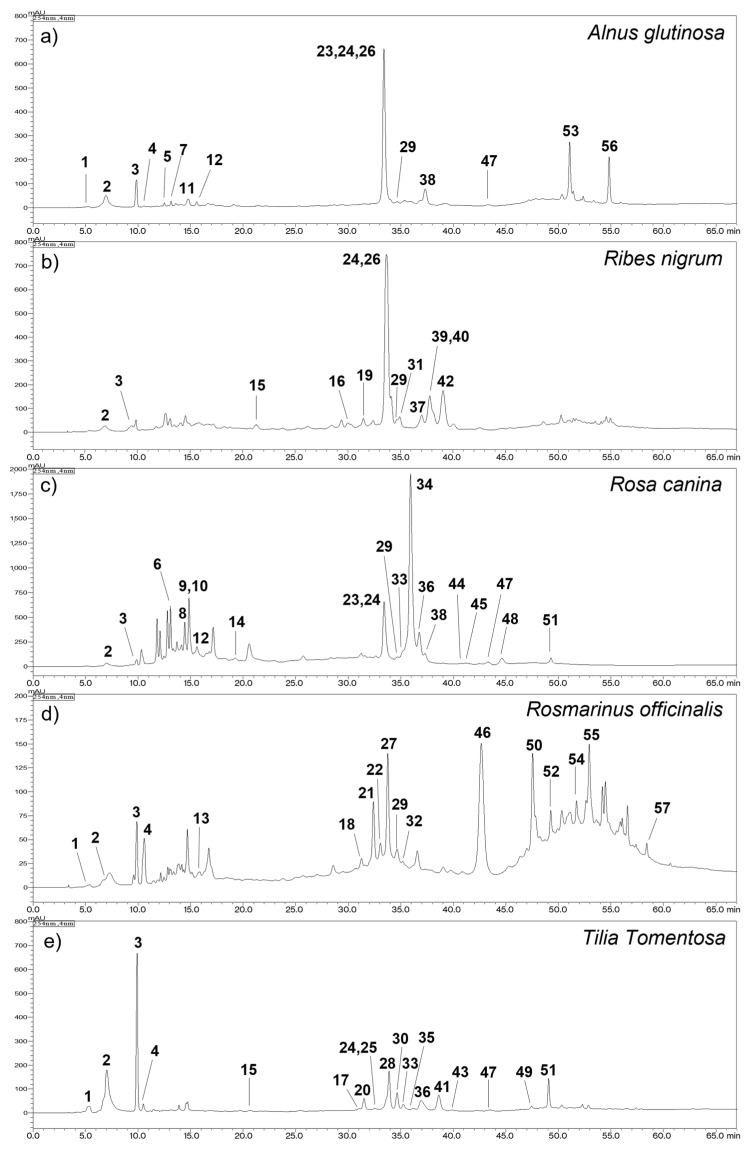
HPLC-DAD profiles of (**a**) *Alnus glutinosa*, (**b**) *Ribes nigrum*, (**c**) *Rosa canina*, (**d**) *Rosmarinus officinalis* and (**e**) *Tilia tomentosa* concentrated bud macerates (λ 254 nm): **1** cytidine (1-β-D-ribofuranosyl-cytosine), **2** uridine (1-β-D-Ribofuranosyluracil), **3** guanosine (2-Amino-9-(β-D-ribofuranosyl)-3,9-dihydro-6H-purin-6-on), **4** thymidine (1-(2-Deoxy-β-D-ribofuranosyl)-5-methyluracil), **5** 3-O-caffeoylquinic acid (neochlorogenic acid), **6** methyl-galloyl glucose, **7** *p*-coumaroylquinic acid, **8** digalloylquinic acid 1, **9** trigalloylglucose, **10** digalloylquinic acid 2, **11** 4-O-caffeoylquinic acid (crypto-chlorogenic acid), **12** 5-O-caffeoylquinic acid (chlorogenic acid), **13** medioresinol, **14** gallotannin, **15** (E)-p-coumaric acid, **16** myricetin-3-O-hexoside, **17** apigenin pentosyl hexoside, **18** hesperetin-7-O-rutinoside (hesperidin), **19** phloretin-2′-O-glucoside (phloridzin), **20** quercetin rhamnosyl hexoside, **21** rosmarinic acid, **22** hispidulin-7-b-glucoside (homoplantaginin/tectoridin), **23** quercetin glucuronide, **24** quercetin-3-O-glucoside (isoquercetin), **25** quercetin-3-O-galactoside (hyperoside), **26** quercetin-3-O-rutinoside (rutin), **27** isorhamnetin-3-O-hexoside, **28** kaempferol rhamnosyl hexoside, **29** caffeic acid ethylester ((E)-ethyl caffeate), **30** quercetin-3,7-O-dirhamnoside, **31** quercetin 3-glucosyl-(1->2)-glucoronide, **32** apigenin-7-O-glucoside (apigetrin), **33** quercetin pentoside, **34** galloyl quercetin glycoside, **35** apigenin 7-O-glucoronide, **36** quercetin 3-O-rhamnoside (quercitrin), **37** kaempferol hexoside, **38** kaempferol glucuronide, **39** isorhamnetin hexoside, **40** kaempferol rhamnosyl-hexoside, **41** kaempferol-3,7-O-dirhamnoside (kaempferitrin), **42** isorhamnetin rutinoside 1, **43** quercetin hexoside, **44** galloyl kaempferol hexoside or hexoside derivative, **45** kaempferol pentoside, **46** luteolin-7-O-glucoronide, **47** kaempferol rhamnoside, **48** quercetin, **49** acacetin 7-O-rutinoside (linarin/acaciin), **50** isorhamnetin, **51** kaempferol-3-O-(coumaroyl)-glucoside (trans-tiliroside), **52** isorhamnetin-rutinoside 2, **53** centaureidin, **54** dihydroxy-dimethoxyflavone 1, **55** rosmanol quinone, **56** dihydroxy-dimethoxyflavone 2 and **57** rosmanol isomer (epiisorosmanol).

All five CBMs were analyzed through HPLC-DAD-MS^2^ metabolic profiling (Figure 2) allowing the identification of 57 different compounds (Table 2). Metabolites were distributed in 8 chemical classes: flavonoids, nucleosides, phenolic acids, gallotannins and galloyl flavonol glycosides, glycosylated dihydrochalcones, lignans, quinones and abietane type diterpenes. Most of these classes were previously described in bud macerates, except nucleosides. Indeed, four nucleosides were identified here: cytidine **1** (*Tt*), uridine **2** (*Rn*, *Rc*, *Tt*), guanosine **3** (*Rn*, *Rc*, *Tt*) and thymidine **4** (*Tt*). They were previously described in *Cordyceps* samples [25].

#### 2.2.1. Composition of CBM Obtained from *Alnus glutinosa* (*Ag*)

*Alnus glutinosa* CBM is represented by coumaroyl **7** and caffeoyl quinic acids (**5**, **11**, **12**), quercetin (**23**–**24**, **26**) and kaempferol (**38**, **47**) glycosylated flavonols, the flavonol aglycone centaureidin (**53**) and the flavone aglycone dihydroxy-dimethoxyflavone 2 (**56**). Glycosides of quercetin were described before in buds of *Ag* by Peev et al. [27]. Kaempferol-di-desoxyhexoside was isolated in leaves and bark of another *Betulaceae*, *Corylus maxima* [31]. Ferulic and *p*-coumaric acids, esters of quinic acid as well as hyperoside were described as compounds of *Ag* buds but not identified in our CBM [27]. Other phenolic compounds, such as diarylheptanoids (ex: oregonin) were often described in *Ag* [35], *Alnus* species [36,37] or other *Betulaceae* (*Corylus avellana* and *maxima*) [31,38] as well as tannins with a galloyl group [37] or condensed tannins [36], but these compounds were not found in this work. Aglycones, centaureidin and dihydroxy-dimethoxyflavone 2 are described here for the first time in *Ag* glycerin macerate. Centaureidin **53** was firstly reported in *Achillea millefolium* (*Asteraceae*, aerial parts) [32] whereas dihydroxy-dimethoxyflavone 2 **56** such as cirsimaritin is common in *Lamiaceae* species such as *Rosmarinus officinalis* (leaves) [33,34]. Apolar compounds (ELSD) were not further investigated in our study but are most probably associated with terpenoids and steroids as described by Felföldi-Gava et al. [39] and Ren et al. [37].

#### 2.2.2. Composition of CBM Obtained from *Ribes nigrum* (*Rn*)

*Ribes nigrum* CBM was characterized by the presence of (E)-*p*-coumaric acid **15**, glycosylated flavonols exhibiting quercetin (**24**, **26**, **31**), kaempferol (**37**, **40**), myricetin **16** or isorhamnetin (**39**, **42**) as aglycone, caffeic acid ethyl ester **29** together with the glycosylated dihydrochalcone phloridzin **19**.

Coumaric acid, rutin and isoquercitrin were already described in *Rn* glycerin macerates in the literature [11,13,18]. The same authors evidenced the presence of benzoic acids, catechins, other cinnamic acids, other flavonols, terpenic compounds, vitamins and organic acids [9,13,14,15,18]. In 2015, Ieri et al. [16] published the phenolic composition of “bud extracts” of *Rn* which is in accordance with our work. No author noticed the presence of phloridzin in bud macerate, but it was described as a phenolic compound from blackcurrant fruit [29].

#### 2.2.3. Composition of CBM Obtained from *Rosa canina* (*Rc*)

*Rosa canina* CBM contained chlorogenic acid **12**, five gallotannins derived from gallic or galloylquinic acid (**6**, **8**–**10**, **14**) in association with two glycosylated galloyl flavonols: a quercetin one **34** and a kaempferol one **44** and eight glycosylated flavonols derived from quercetin **23**–**24**, **33**, **36** and kaempferol **38**, **45**–**47**, **51**, caffeic acid ethyl ester **29** together with quercetin **48**.

These results are in accordance with Ieri et al. [16] who described glycosides of quercetin and kaempferol, gallic acid derivatives and caffeoylquinic acids together with ellagic acids derivatives, these last compounds being absent of the extracts analyzed here. Kaempferol galloyl hexoside derivative **44** and quercetin **48** are described for the first time in a *Rc* bud macerate. These compounds were identified respectively by Riffault et al. [26] in *Rosa hybrida* and Ozcan et al. [40] in *Rosa canina* alcoholic extracts.

#### 2.2.4. Composition of CBM Obtained from *Rosmarinus officinalis* (*Ro*)

*Rosmarinus officinalis* CBM analysis led to the identification of a great diversity of compounds: one lignan (medioresinol **13**), one phenolic acid (rosmarinic acid **21**), six glycosylated flavonoids: one flavanone (hesperetin **18**), three flavones (hispidulin **22**, apigenin **32**, and luteolin **46**) and two flavonols (glycosides of isorhamnetin **27** and **52**), two aglycone flavonoids: one flavonol (isorhamnetin **50**) and one flavone (di-hydroxy-di-methoxy-flavone **54**), one quinone (rosmanol quinone **55**) and one abietane-type diterpene (epiisorosmanol **57**). Unlike other macerates, no derivative of quercetin or kaempferol could be observed. The presence of luteolin, hesperetin and hispidulin glycosylated flavonoids corroborates Del Bano et al. [41] work on *Rosmarinus officinalis* young shoots extract. Some polyphenolic compounds described in other types of extracts did not appear in our preparations such as carnosic acid [28,30] and diosmin [41].

#### 2.2.5. Composition of CBM Obtained from *Tilia tomentosa* (*Tt*)

*Tilia tomentosa* CBM was characterized by the presence of (E)-*p*-coumaric acid **15**, glycosylated flavonols and flavones derived from quercetin (**20**, **24**–**25**, **30**, **33**, **36**, **43**), kaempferol (**28**, **41**, **47**, **51**), apigenin (**17**, **35**) and acacetin **49**.

All these compounds were already described by Ieri et al. [16]. Peev et al. exhibited in foliar bud glycerin macerate of *Tt*, chlorophylls, carotenoids, provitamin A, polyphenols, vegetal hormones (auxin, cytokinin and gibberellin) in addition to saponins [19].

### 2.3. Highlight on Flavonoid Content of CBMs

Since flavonoids (glycosylated and aglycones) were identified as major constituents of the glycerin macerates, they were quantified at 355 nm as rutin equivalents (RE mg/L) for comparison purpose (Table 3).

This study revealed *Rc* as the most concentrated macerate (809 RE mg/L) whereas other extracts varied from 175 to 470 RE mg/L at 355 nm.

The lack of homogeneity in flavonoid quantification protocols and results expression makes comparisons challenging. Therefore, the flavonoid contents of the five studied plants were determined as mg of rutin equivalent (RE) per L of glycerin macerate, and then calculated per g of dry weight (DW) and per g or 100 g of fresh weight (FW) and exposed in Table 3 to compare with literature data.

For *Ag*, the flavonoid content was evaluated as 470 ± 6 mg RE/L equivalent to 9 mg RE/g of dry weight (DW). This could be compared to Dahija et al. [42] results, exploring the flavonoid contents of methanolic extracts of *Ag* leaves and showing 11.8 mg of rutin equivalent/g of DW (415 nm).

Flavonoid content of CBM obtained from *Rn* (175 ± 2 RE mg/L) is in the range of the description of *Rn* bud macerates analyzed by Ieri et al. [16]: 67–304 mg/L. Expressed in term of fresh weight, a flavonoid content of 1 RE mg/g of FW is below the one described by Liu et al. [24] who evaluated 3–4 mg/g but a different extraction solvent was used (water/acetone). Expressed in mg/g of fresh weight we found a value (150 mg/100 g) close to the one described by Donno et al. [14]: 126 mg/100 g and Turrini et al. [10]: 97 mg/100 g (calculated as the sum of the quercetin, quercitrin and rutin equivalents).

Flavonoid quantification for *Rc* showed a great amount of 809 ± 13 mg RE/L which was higher than that found by Ieri et al. [16] who calculated the amount of flavonol glycosides and evaluated it as 238–589 mg/L, depending on the farm (month and year of harvest).

*Ro* showed the third higher flavonoid content after *Rc* and *Ag* with 332 ± 2 mg RE/L corresponding to 7 mg/g of dry buds (DW). These results could be compared to the flavones concentration determined to be 5.2 mg/g of DW leaves in methanolic extracts [43]. Besides glycosylated and aglycones flavones, we found glycosylated and aglycone flavonols, plus glycosylated flavanone. A higher total flavonoid content of 24.6 mg RE/g of dry leaves (DW) extract (hexane then ethyl acetate) was reported by Kontogianni et al. [30]. These organic solvents could probably permit the enhancement of the extraction yield of *Ro*.

Flavonoid content for *Tt* corresponded to 219 ± 2 mg RE/L of glycerin macerate. This value was in accordance with those determined on flavonol glycosides by Ieri et al. [16] on 5 different buds of *Tt* glycerin macerates: 176–480 mg/L. It could be noted that in our work, besides glycosylated flavonols, we also detected glycosylated flavones. Another work of Turrini et al. [6] evaluated the flavonol content of *Tt* glycerin macerates at 52–91 mg/100 g of fresh weight depending on the process used. Our results showed a similar value (92 mg/100 g of fresh weight) even if no quercetin was detected in our macerate.

### 2.4. Antioxidant Activity

**Figure 3:**
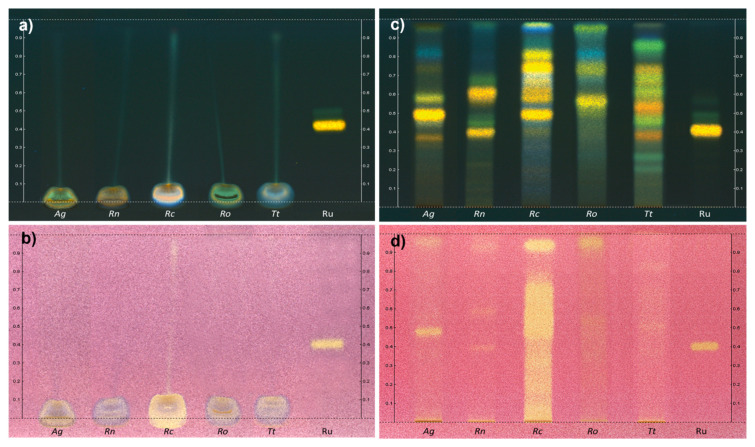
TLC profiles of the five CBMs respectively with Neu reagent and DPPH solution before SPE (**a**,**b**) and after SPE (**c**,**d**) (*Ag*: *Alnus glutinosa*, *Rn*: *Ribes nigrum*, *Rc*: *Rosa canina*, *Ro*: *Rosmarinus officinalis*, *Tt*: *Tilia tomentosa*, Ru: rutin as standard).

An HPTLC study was undertaken using Neu (flavonoids) and DPPH (antioxidant activity) as revelation reagents. CBMs were developed before (Figure 3a,b) and after (Figure 3c,d) SPE preparation. It was observed that glycerol severely hampered the TLC migration and *Rc* exhibited, as expected, the most interesting antioxidant profile (Figure 3d).

DPPH and ORAC experiments were also performed on the five glycerin macerates. These assays gave different results, varying, respectively, from <200 to 4857 µmol TE/g and from 2487 to 6479 µmol TE/g (see Table 4), but they both confirmed the highest antioxidant activity of *Rc* (4857 and 6479 µmol TE/g of dry matter, respectively, using DPPH or ORAC assays).

The DPPH antioxidant activity seemed to be positively correlated with the flavonoid content. The same trend is not observed for the ORAC assay. The major difference in the chemical composition of *Rc* was the presence of galloyl quinic derivatives and galloyl flavonol glycosides. Several works showed strong antioxidant activities for gallic acid and galloyl derivatives. Furthermore, the presence of one or more galloyl moieties was correlated with the antioxidant capacity of flavonol glycosides and galloyl quinic derivatives [44,45,46].

Only a few works describing antioxidant activities of *Rn* and *Ag* bud macerates, [5,12,18] were related in the literature, but the results are not comparable. On the other hand, the antioxidant activities of *Ro* and *Rc* were studied, but they concerned extracts obtained using different solvents of extraction [30,47,48].

## 3. Materials and Methods

### 3.1. Chemical Reagents and Materials

Water was purified with a milli-Q system. Methanol was provided from Honeywell (Seelze, Germany). The 6-Hydroxy-2,5,7,8-tetramethylchroman-2-carboxylique acid (Trolox^®^), formic and chlorogenic (5-O-caffeoylquinic acid) (99%) acids were provided by Acros (Morris Plains, NJ, USA). The *p*-coumaric (98%) and rosmarinic (98%) acids, hesperidin (hesperetin-7-O-rutinoside) (80%), quercetin (98%) and rutin-hydrate (quercetin-3-O-rutinoside) (94%) were provided by Sigma (Steinheim, Germany). Hyperoside (quercetin-3-O-galactoside) (98%) was provided by Extrasynthese (Genay, France). The 1,1-Diphenyl-2-picrylhydrazyl (DPPH) was purchased from Sigma Aldrich (L’Isle d’Abeau Chesnes, France). The 2,2′-Azobis (2-methylpropionamidine) dihydrochloride (AAPH) and fluorescein (FL) were obtained from Acros Organics (Noisy-Le-Grand, France). The following natural products were present in the chemical library of the SONAS laboratory: neo-chlorogenic acid (3-O-caffeoylquinic acid) (83%) isolated from the petals of *Hydrangea*, centaureidin (50%) from *Alnus glutinosa* young shoots, linarin/acaciin (acacetin-7-O-rutinoside) (96.4%), trans-tiliroside [kaempferol-3-O-(coumaroyl)-glucoside] (76.1%) and thymidine (62.4%) from *Tilia tomentosa* buds.

### 3.2. Samples

#### 3.2.1. Concentrated Bud Macerates (CBMs)

CBMs of young shoots or foliar buds of *Alnus glutinosa* (L.) Gaertn, *Ribes nigrum* L., *Rosa canina* L., *Rosmarinus officinalis* L. and *Tilia tomentosa* M. collected in 2017 or 2018 (Table 5) were freshly prepared in a mixture of water/ethanol 96% v/v/glycerol (1/1/1, *v*/*v*/*v*) by a 21-day maceration at room temperature, daily manually mixed, then filtered. Young shoots or foliar buds were freshly incorporated without any grinding at a concentration of 5% of dried matter in the solvent mixture (m/v). In order to evaluate the dry matter, the humidity level was calculated according to the European Pharmacopeia protocol [1]. (*Alnus glutinosa* L. Gaertn: 65.7%, *Ribes nigrum* L.: 57.5%, *Rosa canina* L.: 73.8%, *Rosmarinus officinalis* L.: 51.1% and *Tilia tomentosa* M.: 79.0%) (*n* = 1).

#### 3.2.2. Solid Phase Extraction

A solid phase extraction (SPE) procedure was developed and applied to CBMs to remove glycerol. Ethanol was firstly removed from the macerate by evaporation under vacuum. SPE was carried out on a C18 column (500 mg/2.8 mL) (Thermo Scientific, Cardiff Valley Road, TN, USA), activated by methanol and balanced with water. Samples were then adsorbed on the column (2 mL of CBM corresponding to 100 mg of dried plant) and the glycerol was eluted with water. Compounds adsorbed on the C18-column were recovered by elution with methanol. This solution was then evaporated under vacuum to obtain the final dry extract. These extracts were used to calculate extraction yields, expressed in percentage of the CBM (5% of dried plant: 5 g in 100 mL of solvent (water/ethanol/glycerol (1/1/1, *v*/*v*/*v*)) (*n* = 3).

### 3.3. Phytochemical Analysis

#### 3.3.1. HPLC-DAD-ELSD

Chromatographic analyses were carried out using a 2030C 3D liquid chromatograph equipped with a DAD detector (Shimadzu Corporation, Kyoto, Japan) and an ELSD detector (SEDERE TT-ELSD, Alfortville, France) with a C18, 250 × 4.6 mm, 5 μm column (Macherey-Nagel, Düren, Germany). The mobile phase was constituted by (A) acidified water (0.1% of formic acid (FA)) and (B) acidified methanol (0.1% FA). The following gradient was applied: 1% of B (0–3 min), 1–30% of B (3–10 min), 30% of B (10–20 min), 30–45% (20–30 min), 45% (30–40 min), 45–100% of B (40–60 min) and 100% of B (60–67 min) with a flow rate of 1 mL/min and an oven temperature of 20 °C. UV-vis spectra were recorded in the range of 190–600 nm, and chromatograms were acquired at 254 nm. ELSD was thermostated at 30 °C, and a gain of 9 was applied on the signal. CBMs were diluted 1/1 *v/v* with methanol, centrifuged at 13,000× *g* for 10 min and filtered through a 0.45 μm PTFE membrane syringe filter prior to injection (20 μL), to remove traces of suspended materials.

#### 3.3.2. HPLC-UV-ESI-MS^2^

Analytical HPLC-UV-ESI-MS^2^ was run on a 2695 Waters (Guyancourt, France) coupled with a diode array detector 2996 Waters. Column, mobile phases and gradient were the same as previously described for HPLC-DAD-ELSD. Chromatograms were acquired at 254 nm. The mass analyses were performed on a Brucker (Bremen, Germany) ESI/APCI Ion Trap Esquire 3000+ in both positive and negative modes, with the conditions as follows: collision gas, He; collision energy amplitude, 1.3 V; nebulizer and drying gas, N2, 7 L/min; pressure of nebulizer gas, 30 psi; dry temperature, 340 °C; flow rate, 1.0 mL/min; solvent split ratio 1:9; scan range, *m*/*z* 100–1200. Extracts obtained after SPE were prepared in methanol, at a concentration of 10 mg/mL.

### 3.4. Flavonoid Content

The quantification of flavonoids was performed by HPLC-DAD using 7-point regression curves in triplicate. The column, mobile phases and gradient were the same as previously described for HPLC-DAD-ELSD. Chromatograms were acquired at 355 nm. Statistical analysis by R software validated a linear model (r^2^ = 0.9999) with a confidence interval of 95% (Line equation y = 35,662,581x with a R^2^ = 0.9999). CBMs were diluted in methanol (1/1 *v*/*v*), centrifugated, then filtered (0.45 µm, PTFE membrane). Total flavonoid content (TFC) was expressed in milligrams of rutin equivalent (RE) per liter (mg RE/L) of CBM. Total flavonoid amounts are expressed as the mean of three samples ± SD (*n* = 3). Considering the extraction yield (m/v %), the TFC could be calculated in mg RE/g of dry weight (DW). Considering the humidity level (%), the TFC could be calculated in mg RE/g or/100 g of fresh weight (FW).

### 3.5. Antioxidant Activity

#### 3.5.1. HPTLC flavonoids and DPPH revelations

20 µL of CBMs before and after SPE treatment (10 mg/mL), were deposited on four pre-coated silica gel TLC plates Si60 F254 (10 × 20 cm) (Merck, Darmstadt, Germany) using the CAMAG^®^ Automatic TLC sampler 4 (Chromacim, Moirans, France). Rutin was also deposited as the standard compound (20 µL at 1 mg/mL). After migration with the elution system, ethyl acetate/formic acid/glacial acetic acid/water (100/11/11/27) adapted to flavonoids [49], two plates were sprayed with Neu reagent-revealing flavonoids and the others were revealed using a 0.2% m/v methanolic DPPH (2,2-diphenyl-1picrylhydrazyl) solution for evidence of radical scavenging activity. Pictures of plates were taken using CAMAG^®^ TLC visualizer (Chromacim, Moirans, France) and Wincats software.

#### 3.5.2. Scavenging Activity of diphenyl-picrylhydrazyl (DPPH) Radicals

The diphenylpicrylhydrazyl (DPPH) radical scavenging evaluations of the CBMs were performed as previously described [50]. In its radical form, DPPH• has an absorption band at 517 nm, which disappears upon reduction by an antiradical compound. Briefly, the tested samples and standards were diluted in absolute EtOH at 0.02 mg/mL from stock solutions at 1 mg/mL in DMSO (depending on the yields). Aliquots (100 μL) of these diluted solutions were placed in 96-well plates in triplicate. A total of 25 μL of freshly prepared DPPH solution (1 mM) were added to 75 μL of absolute EtOH using the microplate reader’s injector (Infinite 200, Tecan, France) to obtain a final volume of 200 μL per well. After 30 min in the dark and at ambient temperature, the absorbance was determined at 517 nm. EtOH was used as a blank, and 10, 25, 50, and 75 μM solutions of trolox (hydrophilic α-tocopherol analog) were used for the calibration curve. Samples of chlorogenic acid ethanolic solution and rosemary ethanolic extract (both at 0.02 mg/mL) were used as the positive control standard. Results were expressed as trolox equivalents (micromoles of TE per gram of dry matter).

#### 3.5.3. Measurement of Oxygen Radical Absorbance Capacity (ORAC)

ORAC assays were carried out on CBMs according to the method described by Huang et al. [51] with some modifications. This assay measures the ability of antioxidant compounds to inhibit the decline in fluorescein (FL) fluorescence that is induced by a peroxyl radical generator, 2,2′-azobis (2-methylpropionamidine) dihydrochloride (AAPH). The assay was performed in a 96-well plate. The reaction mixture contained 100 μL of 75 mM phosphate buffer (pH 7.4), 100 μL of freshly prepared fluorescein (FL) solution (0.1 μM in phosphate buffer), 50 μL of freshly prepared 2,2′-azobis(2-methylpropionamidine) dihydrochloride (AAPH) solution (51.6 mg/mL in phosphate buffer), and 20 μL of sample per well. CBMs were analysed in triplicate and diluted in phosphate buffer at different concentrations (25, 12.5, 6.25 and 3.12 μg/mL) from stock solutions at 1 mg/mL in DMSO (depending on the yields). The FL, phosphate buffer, and samples were preincubated at 37 °C for 10 min. The reaction was started by the addition of AAPH using the microplate reader’s injector (Infinite^®^ 200, Tecan, France). Fluorescence was then measured and recorded for 40 min (λexc 485 nm, λem 520 nm). The 75 mM phosphate buffer was used as a blank, and 12.5, 25, 50, and 75 μM solutions of Trolox were used as calibration solutions. A chlorogenic acid solution (8.8 μM) and a rosemary ethanolic extract (12.5 µg/mL) in phosphate buffer were used as positive control standard. The final ORAC values were calculated using a regression equation between the trolox concentration and the net area under the FL decay curve and were expressed as micromole of trolox equivalents per gram of dry matter. Areas under curves were calculated using Magellan data analysis software (Tecan, France).

## 4. Conclusions

In this research, an optimized method was used for the phytochemical analysis of concentrated bud macerates. A preliminary step of solid phase extraction was applied before recording HPLC-DAD-ELSD profiles, HPLC-UV-ESI-MS^2^ analysis and HPTLC revelation, allowing high resolution fingerprints. The phytochemical analysis of *Ag*, *Rn*, *Rc*, *Ro* and *Tt* CBMs led to the identification of 57 compounds distributed in 8 chemical classes: flavonoids, nucleosides, phenolic acids, gallotannins and galloyl flavonol glycosides, glycosylated dihydrochalcones, lignans, quinones and abietane type diterpenes Although phenolic acids and flavonoids are common in all macerates, some compounds are defined particularly in a plant such as a dihydrochalcone **19** in *Rn*; gallotannins (**6**, **8**–**10**, **14**) and galloyl flavonol glycosides (**34** and **44**) in *Rc*; and an abietane-type diterpene **57**, a lignan **13** and a quinone **55** in *Ro*.

Moreover, the phytochemical analysis of CBMs of *Ag*, *Rc*, *Rn* and *Tt* was in agreement with previously reported data and the chemical composition of *Ro* CBMs was investigated for the first time. This study highlighted a great chemical diversity, which is in accordance with the traditional description of bud macerates. Strong antioxidant activities, especially for *Rc* CBM, also support their use in gemmotherapy.

## Figures and Tables

**Table 1:** Bud macerates related in the literature.

Latin Name (Genus and Species)	Common Name	Plant Parts	Reference(s)
*Alnus glutinosa* (L.) Gaertn	Alder	Buds	[5]
*Carpinus betulus* L.	Hornbeam	Buds	[5,6]
*Castanea sativa* Mill. and *Castanea crenata* Siebold and Zucc.	Sweet and Korean chestnut	Buds	[7,8,9,10]
*Cornus mas* L.	Cornelian cherry	Buds	[6]
*Corylus avellana* L.	Hazelnut	Buds	[7,9]
*Ficus carica* L. and SSP *Dottato*	Fig	Buds	[5,6,11]
*Fraxinus excelsior* L.	European ash	Buds	[6]
*Juglans regia* L.	Walnut	Buds	[7,9]
*Larix decidua* Mill.	European larch	Buds	[6]
*Pinus montana* Mill.	Mountain pine	Buds	[6]
*Quercus petraea* (Matt.) Liebl.	Sessile oak	Buds	[6]
*Ribes nigrum* L.	Blackcurrant	Buds	[5,7,9,12,13,14,15,16,17,18]
*Rubus idaeus* L.	Raspberry	Buds	[9]
*Rubus ulmifolius* L. or Schoot	Blackberry	Buds or sprouts	[7,9,14,15]
*Rosa canina* L.	Dog rose	Buds and young sprouts	[16]
*Salix caprea* L.	Willow	Buds	[9]
*Tilia tomentosa* Moench.	Silver Lime	Buds, quiescent buds and sprouts	[6,16,17,19]
*Tilia vulgaris* Hayne	Linden	Buds	[7,9]
*Vitis vinifera*	Grape vine	Buds	[12,17]

**Table 2:** HPLC/UV (254 nm) and MS1, MS2 data obtained after negative and positive ionization of CBMs of *Alnus glutinosa* (*Ag*), *Ribes nigrum* (*Rn*), *Rosa canina* (*Rc*), *Rosmarinus officinalis* (*Ro*) and *Tilia tomentosa* (*Tt*).

N°	Plants	Rt (min)	λ_Max_ (nm)	[M+H]^+^ /[M-H]^−^ (*m*/*z*)	Fragments +/− (*m*/*z*)	MS2^+^/MS2^−^ (*m*/*z*)	MW (g/mol)	Compounds * Identification Method	Ref
1	*Ag*, *Ro*, *Tt*	5.3	279	244/242	112/110	112/190,152,110	243	Cytidine	[25]
2	*Ag*, *Rn*, *Rc*, *Ro*, *Tt*	6.9	261	-/243	113/200,111	-/200,152,140,110	244	Uridine	[25]
3	*Ag*, *Rn*, *Rc*, *Ro*, *Tt*	9.9	252	284/282	152/150	152,135/150,133	283	Guanosine	[25]
4	*Ag*, *Ro*, *Tt*	10.6	261	-/241	127/-	-/223,198,151,125	242	Thymidine **	[25]
5	*Ag*	12.5	217,238,300,325	-/353	-/-	-/191,179,135	354	Neo-chlorogenic acid *	[16]
6	*Rc*	13.1	221,267	347/345	185/-	-/183,124	346	Methyl-galloyl glucose	[16]
7	*Ag*	13.7	224,310	339/337	147/163	-/191,173,163,119	338	*p*-Coumaroyl quinic acid	[16]
8	*Rc*	14.4	221,278	-/495	-/635	-/343,169	496	Digalloylquinic acid 1	[16]
9	*Rc*	14.6	278	-/635	467/495	-/465,421,313	636	Trigalloylglucose	[16,26]
10	*Rc*	14.8	222,275	497/495	-/635	-/343,169	496	Digalloylquinic acid 2	[16]
11	*Ag*	14.8	218,239,299,325	355/353	263,193,163/ 253,173	-/191,179,173,135	354	Crypto-chlorogenic acid	[16]
12	*Ag*, *Rc*	15.6	218,239,298,325	355/353	245,173,163 /279,191	-/191	354	Chlorogenic acid *	[16,26,27]
13	*Ro*	15.7	230, 312	389/387	406,227,209,191/-	-/363,207,163	388	Medioresinol	[28]
14	*Rc*	19.3	269	499/497	432,315/-	485,315,279,153 /465,345,183	498	Gallotannin	[16]
15	*Rn, Tt*	21.4	223,309	-/163	-/119	-/119	164	(E)-*p*-coumaric acid *	[16]
16	*Rn*	30.0	260,358	481/479	319/477,403	319/317,179	480	Myricetin-3-O-hexoside	[16]
17	*Tt*	31.0	271,334	565/563	-/-	-/473,443,383,353	564	Apigenin pentosyl hexoside	[16]
18	*Ro*	31.3	224,283	611/609	449,303,173/-	557,449,369,303/ 301,199	610	Hesperidin *	[28]
19	*Rn*	31.5	222,284	-/435	275/360,273	442,366,296/273,167	436	Phloridzin	[29]
20	*Tt*	31.5	256,355	611/609	-/449	449,303/463,447,301	610	Quercetin rhamnosyl hexoside	[16]
21	*Ro*	32.4	219,284,329	361/359	383,163/179	-/223,197,179,161,133	360	Rosmarinic acid *	[28]
22	*Ro*	33.1	253,348	463/461	-/359	-/285,179,161	462	Homoplantaginin/ Tectoridin	[28]
23	*Ag*, *Rc*	33.4	256,355	479/477	303,167/-	303,167/301,179	478	Quercetin glucoronide	[16]
24	*Ag*, *Rn*, *Rc*, *Tt*	33.4	256,356	465/463	505, 464,302/-	426,303/301,179	464	Isoquercetin	[16,27]
25	*Tt*	33.6	257,353	465/463	-/-	426,303/301,179	464	Hypersoside *	[16]
26	*Ag*, *Rn*	33.7	256,356	611/609	505,465,303/463,373	465,303/301	610	Rutin *	[16,27]
27	*Ro*	33.8	270,346	479/477	-/-	317/315,300	478	Isorhamnetin-3-*O*-hexoside	[28]
28	*Tt*	33.9	265,347	595/593	433/-	-/447,431,285	594	Kaempferol rhamnosyl hexoside	[16]
29	*Ag*, *Rn*, *Rc*, *Ro*	34.7	218,242,298,326	209/207	191,173,163/-	-/179,161,135	208	Caffeic acid ethylester	[16]
30	*Tt*	34.7	256,350	595/593	-/-	-/447,301	594	Quercetin-3,7-*O*-dirhamnoside	[16]
31	*Rn*	34.9	252,357	641/639	519,503/517,377,207	495,478,333,272/331	640	Quercetin 3- glucosyl-(1->2)-glucoronide	[16]
32	*Ro*	35.2	220,294,332	-/431	227,185,173/-	-/269	432	Apigetrin	[28,30]
33	*Rc, Tt*	35.3	258,356	435/433	303,173 /301	-/301	434	Quercetin pentoside	[16]
34	*Rc*	35.9	252,301,366	617/615	504,435,315,303,173 /433,301	-/301	616	Galloyl quercetin glycoside	[16]
35	*Tt*	36.2	266,338	447/445	-/343,269,175	271/269,175	446	Apigenin-7-*O*-glucoronide	[16]
36	*Rc, Tt*	36.9	258,348	449/447	303,173/301	-/301	448	Quercitrin	[16]
37	*Rn*	37.0	264,348	449/447	448,287/377	303,287/301,285,255	448	Kaempferol hexoside	[16]
38	*Ag*, *Rc*	37.3	265,348	463/461	462,303,287/447,301	287/285,175	462	Kaempferol glucoronide	[16,31]
39	*Rn*	37.8	255,353	479/477	478/404	460,317/ 357,315,314,271	478	Isorhamnetin hexoside	[16]
40	*Rn*	38.2	251,266,306,357	-/593	-/447	-/-	594	Kaempferol rhamnosyl- hexoside	[16]
41	*Tt*	38.6	264,343	579/577	433/-	-/431,285	578	Kaempferitrin	[16]
42	*Rn*	39.1	254,296,354	625/623	479,317/507,385	479,317/315,300,271	624	Isorhamnetin rutinoside 1	[16]
43	*Tt*	39.9	268,354	465/463	-/-	-/301	464	Quercetin hexoside	[16]
44	*Rc*	40.7	268,353	601/599	-/-	315,287,209/313,285	600	Galloyl kaempferol hexoside	[26]
45	*Rc*	41.2	263,347	419/417	287,173/-	287/285,255,227	418	Kaempferol pentoside	[16]
46	*Ro*	42.7	268,341	463/461	-/-	287/285	462	Luteolin-7-*O*-glucoronide	[28]
47	*Ag*, *Rc*, *Tt*	43.3	263,344	433/431	287,173/361,343,191	355,287/285,259,255	432	Kaempferol rhamnoside	[16,31]
48	*Rc*	44.6	254,370	303/301	239,173/-	285,257,229,201,165, 137/179,151	302	Quercetin *	[20]
49	*Tt*	47.5	267,333	593/591	-/-	447,285/591,457,283	592	Linarin/Acaciin **	[16]
50	*Ro*	47.5	254,348	317/315	287/285	302/300	316	Isorhamnetin	[28]
51	*Rc, Tt*	49.2	265,316,366	595/593	287,173/447,285	585,309,287,165/ 447,285	594	Trans-tiliroside **	[16]
52	*Ro*	49.3	-	625/623	-/479,433,345	317,302/315,300	624	Isorhamnetin-rutinoside 2	[28]
53	*Ag*	51.1	256,351	361/359	-/329	346,345,328/344,329	360	Centaureidin **	[32]
54	*Ro*	51.7	274,355	315/313	-/-	300,282,254/ 311,298,283	314	Dihydroxy-di methoxyflavone 1	[28]
55	*Ro*	52.9	218,241,306	345/343	-/-	299,271,231,165 /299,284,243,216	344	Rosmanol quinone	[28]
56	*Ag*	54.8	274,334	315/313	-/283	300/298,283	314	Dihydroxy-dimethoxyflavone 2	[33,34]
57	*Ro*	58.4	266,314,408	-/345	715,369,301	-/301,286	346	Epiisorosmanol	[28]

Identified by comparison with * commercial standards or ** with compounds from the SONAS chemical library when available.

**Table 3:** Total flavonoid content (glycosylated and aglycones in rutin equivalent (RE)).

Samples	mg RE/L of Glycerin Macerate	mg RE/g of Dry Weight (DW)	mg RE/g of Fresh Weight (FW)	mg RE/100 g of Fresh Weight (FW)
*Alnus glutinosa* L. Gaertn	470 ± 6	9	3	322
*Ribes nigrum* L.	175 ± 2	3	1	150
*Rosa canina* L.	809 ± 13	16	4	424
*Rosmarinus officinalis* L.	332 ± 2	7	3	325
*Tilia tomentosa* M.	219 ± 2	4	1	92

**Table 4:** Evaluation of the antioxidant activity of the five CBMs using DDPH and ORAC assays (*n* = 3).

CBMs/Standards	DPPH (µmol TE/g)	ORAC (µmol TE/g)
*Alnus glutinosa* L. Gaertn	1027 ± 92	3397 ± 172
*Ribes nigrum* L.	<200	2487 ± 226
*Rosa canina* L.	4857 ± 48	6479 ± 480
*Rosmarinus officinalis* L.	1038 ± 57	4640 ± 292
*Tilia tomentosa* M.	<200	6417 ± 166
*Chlorogenic acid*	3451 ± 84	12174 ± 1008
*Rosmary ethanolic extract*	1494 ± 119	1931 ± 124

**Table 5:** List of analyzed samples.

Material	English Name	Botanic Family	Plant Parts	Month/Year
*Alnus glutinosa* L. Gaertn	Alder	*Betulaceae*	young shoots	June 2018
*Ribes nigrum* L.	Black currant	*Grossulariaceae*	buds	April 2017
*Rosa canina* L.	Dog rose	*Rosaceae*	young shoots	May 2018
*Rosmarinus officinalis* L.	Rosemary	*Lamiaceae*	young shoots	August 2018
*Tilia tomentosa* M.	Linden	*Malvaceae*	buds	July 2018

## Data Availability

Not applicable.
